# Multimodal evidence chain of iron overload, inflammation, and dysfunction: an integrated predictive model of early cardiac injury in pediatric transfusion-dependent *β*-thalassemia

**DOI:** 10.3389/fcvm.2026.1716239

**Published:** 2026-03-04

**Authors:** Panyan Zhou, Caili Li, Xiaomei Gao, Caifen Ye, Mufang Huang, Heng Zhang

**Affiliations:** 1 Department of Ultrasonic Imaging, Zhuhai People’s Hospital (The Affiliated Hospital of Beijing Institute of Technology, Zhuhai Clinical Medical College of Jinan University), Zhuhai, China; 2 Department of Hematology and Rheumatology, Zhuhai People’s Hospital (The Affiliated Hospital of Beijing Institute of Technology, Zhuhai Clinical Medical College of Jinan University), Zhuhai, China

**Keywords:** B-type natriuretic peptide, cardiac dysfunction, iron overload mechanism, transfusion-dependent *β*-thalassemia, troponin I

## Abstract

**Introduction:**

Despite standardized transfusion and chelation therapy, children with transfusion-dependent *β*-thalassemia (TDT) remain at high risk for cardiac dysfunction due to iron overload. Conventional ejection fraction assessment lacks sensitivity for early injury. This study evaluated multimodal indicators to develop a robust early-warning model.

**Methods:**

A prospective cohort of 128 TDT children (3–16 years) underwent cardiac magnetic resonance (CMR) T2* imaging, echocardiography with global longitudinal strain (GLS), and measurement of circulating biomarkers including high-sensitivity cardiac troponin I (hs-cTnI), B-type natriuretic peptide (BNP), interleukin-6, and tumor necrosis factor-α. Children were classified into dysfunction and normal groups based on LVEF and GLS. Logistic regression identified predictors, and ROC analysis validated the integrated model.

**Results:**

The dysfunction group demonstrated reduced GLS, ventricular remodeling, elevated hs-cTnI and BNP, and significantly shorter T2* values compared with controls (*p* < 0.001). Inflammatory cytokines were also upregulated. Multivariate analysis identified hs-cTnI, BNP, and T2* as independent predictors. The combined three-factor model achieved excellent discrimination (AUC 0.914), outperforming single markers, with preserved calibration following bootstrap validation.

**Conclusion:**

By linking iron overload, myocardial injury, inflammation, and structural dysfunction, this study proposes a clinically feasible integrated model for early cardiac risk detection in pediatric TDT. The approach supports precision monitoring and prevention of heart failure.

## GRAPHICAL ABSTRACT

**Figure:**
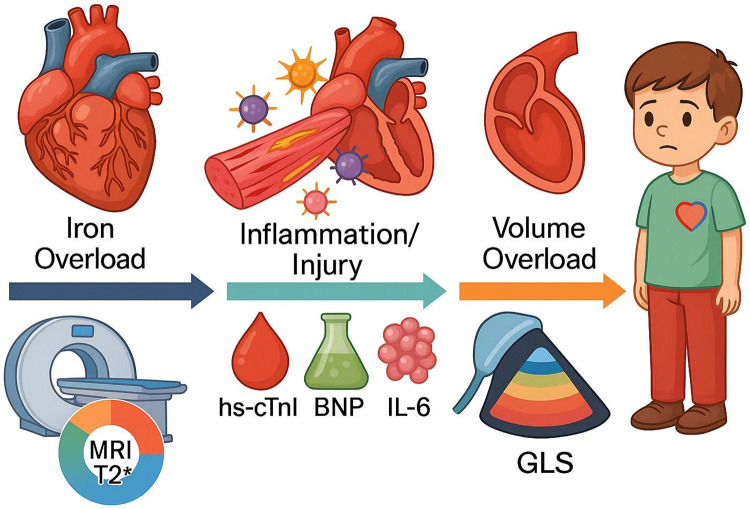
Iron overload drives cardiac dysfunction in children with TDT via inflammatory and myocardial injury pathways.

## Introduction

*β*-Thalassemia is an autosomal recessive disorder characterized by reduced or absent synthesis of *β*-globin chains (1). It is highly prevalent in the Mediterranean, Middle East, South Asia, and Southern China (2). Transfusion-dependent *β*-thalassemia (TDT), the most severe clinical phenotype, typically manifests within months after birth with progressive anemia, growth retardation, skeletal deformities, and hepatosplenomegaly (1, 3).

Although regular blood transfusions and iron chelation therapy (ICT) have significantly improved patient survival, transfusion-associated iron overload remains a critical determinant of long-term prognosis (4, 5). Cardiac iron deposition is one of the leading causes of mortality, accounting for >70% of deaths due to heart failure, arrhythmias, and other cardiovascular complications (6, 7). Iron overload cardiomyopathy often lacks early symptoms, and conventional cardiac function assessments frequently fail to detect subclinical abnormalities, posing challenges for clinical management (8). Notably, left ventricular ejection fraction (LVEF) may remain normal despite early myocardial injury, limiting its sensitivity for early screening.

Recent advances in cardiovascular magnetic resonance (CMR) and echocardiography have provided new approaches for refined cardiac function assessment (9). Among these modalities, multi-echo gradient-echo T2* CMR is currently regarded as the gold standard for the quantitative assessment of myocardial iron deposition. Measurement of myocardial T2* values enables accurate estimation of cardiac iron burden, with T2* values <20 ms indicating moderate-to-severe myocardial iron overload and values <10 ms conferring a high risk of severe iron accumulation. In addition, CMR allows simultaneous evaluation of ventricular wall thickness, chamber dimensions, and other indices of structural remodeling, as well as global and regional systolic and diastolic myocardial function, thereby providing comprehensive and noninvasive imaging evidence of iron overload–related myocardial injury (10, 11). Myocardial strain analysis using 2D speckle-tracking echocardiography (2D-STE) can detect subclinical myocardial dysfunction (12, 13). In *β*-thalassemia populations, studies show parameters like global longitudinal strain (GLS) often become abnormal before LVEF changes, suggesting their potential as early monitoring indicators (8, 14). However, systematic application of strain parameters for risk assessment in children with TDT remains insufficient.

Serum biomarkers are widely used in cardiovascular disease diagnosis and risk stratification, including high-sensitivity cardiac troponin I (hs-cTnI) for myocardial injury, B-type natriuretic peptide (BNP) for volume overload and wall stress, and inflammatory markers, which have been applied for early detection and dynamic monitoring (15, 16). However, their predictive value in pediatric TDT lacks systematic validation, with most existing studies being cross-sectional observations that fail to establish robust evidence chains (17, 18). A key translational challenge remains how to effectively integrate serum biomarkers with imaging parameters to improve both risk identification efficiency and clinical applicability (19, 20).

At the public health level, establishing scientific, early, and cost-effective cardiac monitoring strategies for high-risk pediatric populations is of paramount importance, as it not only impacts outcome improvement but also healthcare resource allocation efficiency. The age-related physiological variations and developmental characteristics of pediatric patients necessitate monitoring strategies that account for dynamic changes in parameters and their clinical applicability. In this context, developing a multidimensional cardiac risk prediction model integrating imaging strain parameters and serum biomarkers may enable earlier risk identification through non-invasive and relatively low-cost approaches in resource-limited settings. Incorporating statistical and machine learning modeling methods such as logistic regression and receiver operating characteristic (ROC) curve analysis could further enhance the model's accuracy and interpretability, facilitating the clinical implementation of precision medicine in this field.

Based on these clinical and population evidences, this study aims to systematically evaluate the correlation between cardiac dysfunction and serum biomarkers in children with TDT, and to develop and validate an integrated early-warning model combining myocardial strain analysis with serum biomarker profiles, thereby providing evidence for proactive and precise cardiac protection strategies.

## Materials and methods

### Study design and ethics statement

This study adopted a prospective, single-center, multidisciplinary observational design to investigate early cardiac dysfunction and predictive modeling in children with TDT. All participants were consecutively recruited from Zhuhai People's Hospital, a tertiary referral center in southern China, between July 2021 and July 2024.

The multidisciplinary framework involved coordinated collaboration among specialized units within the same institution. The Department of Hematology and Rheumatology was responsible for patient diagnosis, transfusion and iron chelation management, and clinical follow-up, while the Department of Ultrasonic Imaging conducted standardized echocardiographic and myocardial strain assessments. Cardiac magnetic resonance imaging and laboratory biomarker analyses were performed using unified protocols to ensure methodological consistency across all participants.

All enrolled patients were included as a single cohort under a unified study protocol; therefore, stratification of sample size by center was not applicable. The collection and use of human samples were approved by our Institutional Medical Ethics Committee [No. 2025-LunShen(Tech)-05]. The study protocol and participant/guardian informed consent materials completed ethical filing. Before enrollment, researchers provided comprehensive information disclosure, and written informed consent was obtained from guardians after full understanding of the study purpose, procedures, and potential risks. Each participant was assigned a unique anonymized identifier post-sampling, with identifiable information managed by an independent data administrator and isolated from the research analysis team.

### Patient recruitment and grouping

The clinical study subjects comprised 128 pediatric patients with TDT hospitalized in our institution between July 2021 and July 2024. All patients were definitively diagnosed with TDT through peripheral blood high-throughput sequencing, covering common HBB mutation types (e.g., CD41/42, IVS-II-654) and meeting national diagnostic guidelines. To ensure consistency in baseline treatment interventions, enrolled patients were required to have received standardized transfusion and ICT for at least 6 months, with treatment regimens developed and implemented by the same hematology expert team. Inclusion criteria: (1) Age 3–16 years (with child assent or guardian consent); (2) Complete datasets including 2D-STE, CMR (T2* sequence), serum samples, and follow-up data; (3) Good treatment adherence without significant interruptions. Exclusion criteria: (1) Comorbid congenital/rheumatic heart disease or structural cardiomyopathy; (2) Recent (within 4 weeks) systemic infection, transfusion reaction, myocarditis, or major surgery; (3) Missing key clinical data, nonstandard sample processing, or significant intervention bias. All participants provided written informed consent before data collection. Clinical data and samples were anonymized using unique identifiers and managed through an encrypted, access-controlled research database to ensure privacy and data security.

To investigate correlations between cardiac dysfunction and myocardial strain parameters/serum biomarkers, patients were stratified based on LVEF and GLS values from 2D-STE, following ASE/EACVI guidelines: (1) Normal cardiac function group (*n* = 66): LVEF ≥ 55% and GLS > −18%; (2) Cardiac dysfunction group (*n* = 62): LVEF < 55% or GLS ≤ −18%. All imaging parameters were independently measured by two radiologists with >5 years' experience. Interobserver reliability was confirmed by an intraclass correlation coefficient (ICC) >0.85, with final values representing averaged measurements. The sample size estimation was based on preliminary pilot data and effect sizes reported in previous studies regarding hs-cTnI and GLS for cardiac function stratification. A two-sided *t*-test model was applied with a significance level of *α* = 0.05 and a statistical power of 0.80. The initial calculation indicated that no fewer than 137 participants were required per group (normal cardiac function vs. impaired cardiac function). To account for potential sample loss and incomplete data, we originally planned to enroll 150 children in each group, resulting in a total intended sample size of 300. Ultimately, 128 children were included in the final analysis (66 in the normal group and 62 in the impaired group), which was lower than the planned sample size. The main reasons were as follows: (1) After strict application of exclusion criteria, only 142 cases from 2021 to 2024 met the requirements of “≥6 months of continuous standardized therapy plus complete multimodal imaging data”. Among these, 14 were further excluded due to suboptimal image quality (ultrasound tracking success rate <70% or CMR T2-fitting failure). (2) Regional pandemic control measures during the study period led to disrupted follow-up or delayed examinations in some children, resulting in missing key data. *post-hoc* assessment showed that the final cohort of 128 children remained well balanced between groups in baseline characteristics, including age, sex, disease duration, and treatment regimen (all *p* > 0.05). Furthermore, validation using G*Power 3.1 confirmed that the current sample size still provided sufficient statistical power (0.78) for detecting the primary outcome—namely, the correlation between GLS and hs-cTnI—which is close to the predefined power of 0.80 and meets the requirements for statistical analysis. Baseline comparability tests were performed for key demographic and clinical variables, including age, sex, disease duration, and treatment regimen, to ensure the scientific validity and appropriateness of between-group comparisons.

### Echocardiographic examination and functional measurements

All 2D-STE examinations were performed under resting conditions in a temperature-controlled, quiet ultrasound room, with patients resting supine for ≥10 min to stabilize heart rate and respiration. Using a GE Vivid E95 system equipped with an M5Sc-D cardiac probe (frequency: 1.5–4.6 MHz), two registered sonographer (>5 years' pediatric cardiac imaging experience) conducted standardized examinations. Image acquisition followed the five standard views recommended by ACC/AHA & ASE: parasternal long-axis, short-axis, apical 4-chamber, 2-chamber, and 3-chamber views. All images were stored in DICOM format for analysis, with optimized settings: frame rate 60–90 fps, linear time gain compensation, and depth 12–16 cm to ensure high-fidelity myocardial structural and motion data.

Measured parameters included: LVEF (biplane Simpson's method), left ventricular end-diastolic diameter (LVEDD), interventricular septal thickness (IVSd), left atrial anterior-posterior diameter, right ventricular diameter, and left ventricular mass index (LVMI). Wall motion was semi-quantitatively assessed using WMSI (average of 5 segments). All data were analyzed by two independent radiologists using the EchoPAC (GE Healthcare, v203) platform and read double-blindly. To further improve data consistency, the ICC was used to assess inter-reader agreement, with an ICC value > 0.85 considered highly consistent. If image quality failed to meet the standard due to poor patient compliance or a limited ultrasound window, the case was automatically excluded from the imaging analysis, and a missing value was recorded.

### 2D-STE

Myocardial strain parameters were acquired using 2D-STE technology and analyzed with EchoPAC software (GE Healthcare, version 203). The analysis utilized standard apical 4-chamber, 2-chamber, and 3-chamber views obtained during previous examinations, with frame rates maintained at 60–90 Hz while excluding frames with significant noise, arrhythmias, or drift. Strain analysis included three components: GLS, global circumferential strain (GCS), and global radial strain (GRS). GLS was calculated as the average of endocardial tracking curves from the three apical views, while GCS and GRS were derived from automated tracking of mid-ventricular short-axis views using a 6-segment model. Two board-certified imaging specialists trained in strain analysis independently processed all images under blinded conditions.

To ensure consistency, all strain parameters were measured over three consecutive cardiac cycles, excluding ectopic beats before averaging, with negative values representing myocardial contraction. When automated tracking failed or quality scores fell below 70%, manual adjustment of ROI or endocardial re-tracing was performed. Interobserver agreement was assessed using ICC, with values >0.85 was considered highly consistent. For individual cases with disagreements, a third senior imaging expert reviews and adjudicates the final results. All analyses used anonymized images to prevent measurement bias, and the analysts were unaware of the clinical group information throughout the process. Tracking success rates and quality scores were recorded for each subject and included as covariates in sensitivity analyses to evaluate image quality effects on strain accuracy.

### MRI T2* mapping for myocardial iron assessment

CMR examinations were performed using a Philips Achieva 3.0 T high-field system. Before scanning, all patients received detailed procedural explanations, underwent screening for metal implant contraindications, and provided written informed consent. Subjects rested supine during scanning using dedicated cardiac coils with electrocardiogram-gating and respiratory triggering. The imaging protocol included fast gradient echo T2* sequences for myocardial iron quantification, covering the mid-ventricular short-axis plane with 10 mm slice thickness. Echo times (TE) were sequentially set at 2.6, 4.5, 6.4, 8.3, 10.2, 12.1, 14.0, 15.9 and 17.8 ms, with each TE acquired twice to improve signal-to-noise ratio. Standard acquisition parameters were: repetition time = 22 ms, flip angle = 20°, matrix size = 256 × 128, field of view = 360 mm × 320 mm.

All images were stored in DICOM format and analyzed using cvi42 software (Circle Cardiovascular Imaging, Calgary, Canada) for automated T2* fitting. At the mid-ventricular short-axis level, endocardial and epicardial borders were manually traced, excluding papillary muscles and chamber signals. Three mid-myocardial pixel layers were selected for signal decay fitting using exponential regression (S = S₀·exp(-TE/T2)) to calculate T2* values. To ensure objectivity and consistency, two trained imaging specialists who were blinded to the clinical grouping performed blinded analyses independently. T2* < 20 ms indicated moderate-to-severe iron overload, while <10 ms suggested high-risk iron deposition. ICC > 0.90 was considered highly consistent, with discrepancies >10% resolved by a third senior radiologist. Cases with failed fitting due to arrhythmias or artifacts were recorded as invalid and excluded from analysis. All imaging data were coded and archived in a central database for follow-up.

### Standardized serum sample collection and preservation protocol

All serum samples were collected from fasting patients in the morning to ensure baseline hormonal and metabolic consistency. Venipuncture was performed using disposable vacuum collection systems (BD Vacutainer, USA), with 5 mL peripheral venous blood drawn into anticoagulant-free tubes without vigorous shaking to prevent hemolysis. Samples stood at room temperature for 30 min for complete clotting, then underwent immediate centrifugation at 3,000 rpm for 10 min at 4 °C. Using sterile dual-layer pipette tips, supernatant serum was carefully separated without disturbing the red blood cell layer or clot. Each sample was aliquoted into 1 mL volumes in pre-labeled 2.0 mL low-adsorption EP tubes and immediately transferred to −80 °C ultra-low temperature freezers, avoiding repeated freeze-thaw cycles. All procedures were conducted in pre-cooled biosafety cabinets to minimize protease activity and maintain biological stability.

The entire process from collection to storage was executed by the same trained research team following standardized operating procedures (SOPs) with accompanying electronic case report forms to ensure sample-clinical data correspondence. All samples received unique identification codes and were cross-verified with clinical databases, with key clinical information masked to maintain blinding during analysis. Before testing, all serum tubes were thawed at 4 °C for 4 h for gradual rewarming. To control batch effects, samples were analyzed in randomized order within single experimental runs after one-time thawing. Sample storage, retrieval and testing were documented by dual signatures from designated managers and technicians, with complete audit trails to ensure traceability and result reliability.

### Enzyme-linked immunosorbent assay (ELISA) detection strategy for inflammation and cardiac function-related serum biomarkers

Quantitative analysis of target serum biomarkers was performed using ELISA. Based on literature review and clinical applicability, four functionally representative markers were selected: (1) myocardial injury marker—hs-cTnI; (2) volume overload marker—BNP; (3) inflammatory factors—interleukin-6 (IL-6) and tumor necrosis factor-α (TNF-α). Commercial high-sensitivity ELISA kits (R&D Systems, USA) were used, with batch numbers and expiration dates uniformly recorded. The detection protocol was strictly followed by manufacturer instructions, including: room temperature incubation, standard preparation, sample loading, enzyme conjugation, plate washing, chromogenic reaction, and termination. All assays were completed within standardized timeframes to minimize intra-day variation.

Before testing, all serum samples underwent single-batch thawing. Each analyte was measured in triplicate wells with blank controls, positive controls, and standards. Optical density OD values were read at 450 nm using a BioTek Synergy H1 microplate reader, with 570 nm reference wavelength correction to minimize plate variability. Standard curves were fitted using four-parameter logistic (4PL) regression (R^2^ ≥ 0.98 required). Quality control samples were included in each run, with acceptable coefficients of variation (CV) of <10% intra-assay and <15% inter-assay; otherwise, tests were repeated. Data were normalized to concentration units (pg/mL or ng/mL). Values below detection limits were recorded as 50% of the minimum detectable concentration for statistical analysis. To verify reproducibility, 10% of samples were re-tested, with a Pearson correlation >0.90 required between original and repeat measurements. All raw data underwent dual independent entry and cross-verification to ensure accuracy, completeness, and traceability for modeling analyses.

### Construction and validation of predictive models

To develop an early warning model for cardiac dysfunction in children with TDT, we defined cardiac dysfunction status (0 = absent, 1 = present) as the dependent variable. All variables showing significant associations (*p* < 0.10) in univariate analyses were included as candidate independent variables in the multivariate logistic regression model. We employed stepwise selection to identify independent risk factors, with results expressed as odds ratios (ORs) and 95% confidence intervals (95% CIs). Multicollinearity was assessed using variance inflation factors (VIFs), and variables with VIF > 5 were excluded due to significant collinearity.

Following model development, we evaluated its discriminatory power by constructing ROC curves and calculating the area under the curve (AUC). AUC values were interpreted as follows: 0.5–0.7 (low accuracy), 0.7–0.9 (moderate accuracy), and >0.9 (high accuracy). We also computed sensitivity, specificity, positive predictive value, and negative predictive value to comprehensively assess clinical utility.

To validate model stability, we performed internal validation using bootstrap resampling (1,000 iterations) to obtain bias-corrected AUC estimates and CIs, thereby evaluating potential overfitting. Additionally, we randomly divided the original dataset into training (70%, *n* = 90) and validation (30%, *n* = 38) sets. The training set was used for model development, while the validation set assessed external performance. We compared AUC values and other performance metrics between datasets and generated paired ROC curves to visualize model robustness. All performance indicators were reported separately for both training and validation sets.

### Statistical analysis

All statistical analyses were performed using SPSS Statistics software (Version 26.0, IBM Corp., Armonk, NY, USA). Graphical presentations and correlation plots were generated using GraphPad Prism 9.5 (GraphPad Software, USA) and R language 4.3.2 (R Foundation, Austria) with ggplot2 package.

Continuous variables were first assessed for normality using Shapiro–Wilk test. Normally distributed data with homogeneity of variance were expressed as mean ± standard deviation (x¯ ± SD) and compared using independent samples *t*-test (two groups) or one-way analysis of variance (ANOVA) (three or more groups), with Tukey's HSD *post-hoc* test for significant differences. Non-normally distributed or heteroscedastic data were analyzed using non-parametric tests: Mann–Whitney *U* test (two groups) or Kruskal–Wallis *H* test (multiple groups), with a two-tailed significance level set at *p* < 0.05.

Categorical variables were presented as frequencies and percentages, compared using *χ*^2^ test or Fisher's exact test (for small sample sizes). Correlations were analyzed using Pearson (normal data) or Spearman (ordinal/non-normal data) coefficients, with *p* < 0.05 considered statistically significant.

Multiple serum biomarkers were evaluated for cardiac dysfunction prediction using multivariate logistic regression to develop risk scoring systems. Model performance was assessed by ROC curve analysis, calculating AUC, along with sensitivity, specificity and optimal cut-off points. All key data underwent triple independent experimental/technical replicates with blinded analysis to verify consistency. Significance levels were set at *p* < 0.05, with *p* < 0.01 indicating highly significant differences.

## Results

### Well-balanced key clinical variables between two groups after cardiac function stratification

This study established cardiac function stratification in 128 children with TDT using combined echocardiographic criteria: LVEF ≥ 55% plus GLS > −18% defined the normal cardiac function group (*n* = 66), while LVEF < 55% or GLS ≤ −18% identified the cardiac dysfunction group (*n* = 62). The overall cohort demonstrated a mean age of 10.3 ± 3.2 years with a balanced gender distribution (male:female ratio = 1.14:1) and a high enrollment rate (79.4%).

Comprehensive baseline analysis confirmed excellent intergroup comparability across multiple clinical parameters: age (10.2 ± 3.1 vs. 10.4 ± 3.3 years, *p* = 0.62 by independent *t*-test), gender (*χ*^2^ = 0.06, *p* = 0.94), disease duration (7.4 ± 2.8 vs. 7.6 ± 2.9 years, *p* = 0.58), transfusion frequency (13.1 ± 2.4 vs. 13.3 ± 2.7 sessions/year, *p* = 0.44), ICT (deferoxamine [DFO] plus deferasirox (DFX) combination: 76.7% vs. 78.0%, *p* = 0.77 by *χ*^2^ test), and treatment adherence (visual analog scale [VAS] scores: 8.5 ± 1.2 vs. 8.3 ± 1.4, *p* = 0.33). Socioeconomic parameters, including household income brackets and guardian education levels, showed identical distributions between groups.

**Figure 1:**
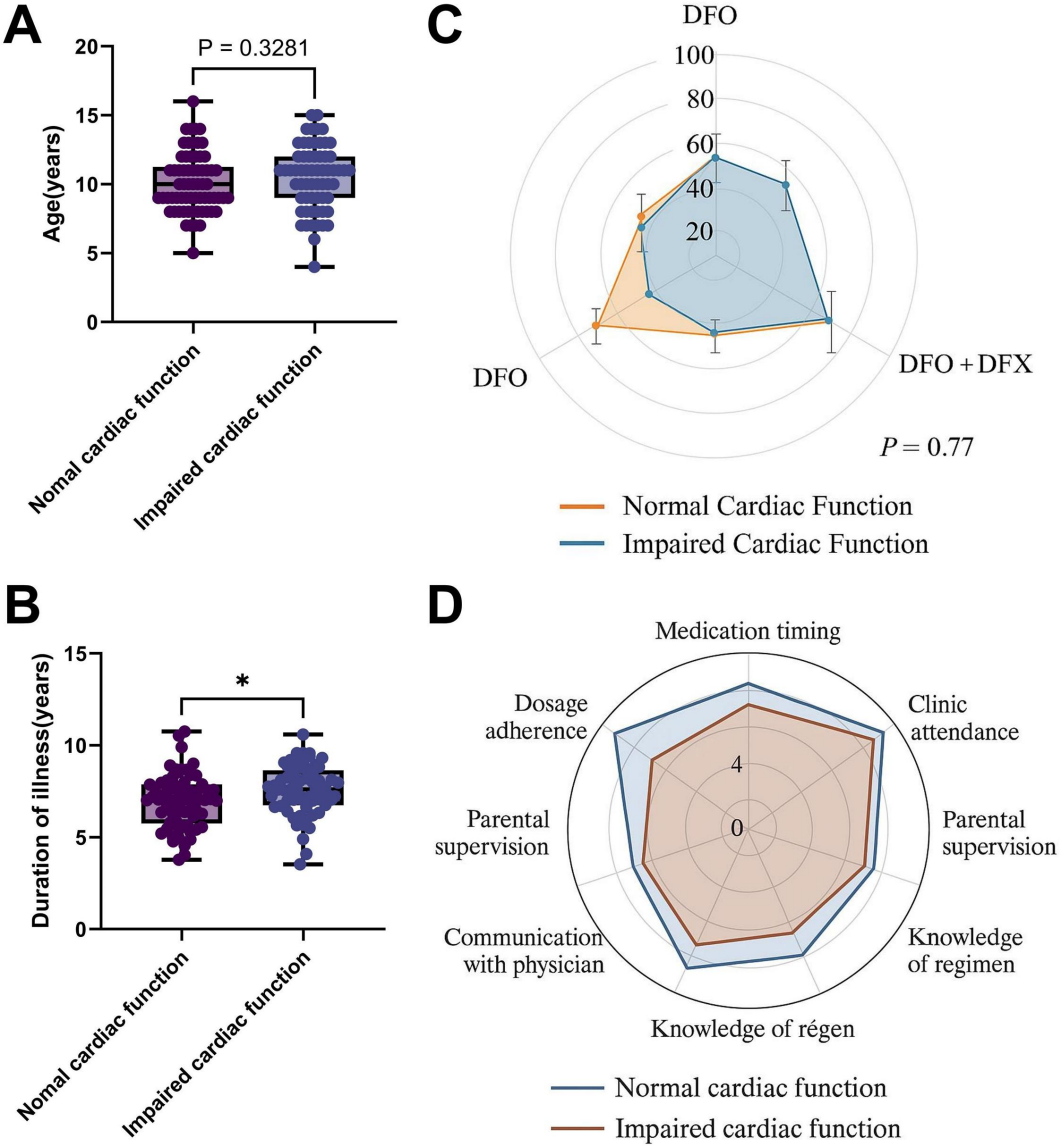
Cohort enrollment flowchart and baseline Variable balance analysis. **(A,B)** Boxplots of age and disease duration distributions demonstrating intergroup overlap (*p* = 0.62 and 0.58, respectively); **(C)** Radar chart of treatment adherence (VAS scores) showing consistent distributions; **(D)** Radar chart of iron chelation regimens (DFO, DFX monotherapy and combination therapy) revealing comparable proportions between groups. Error bars represent SD. All *p*-values were derived from independent *t*-tests or *χ*^2^ tests, with none reaching statistical significance (*p* > 0.05).

Graphical analyses reinforced these findings, with boxplot visualization of age and disease duration demonstrating complete distributional overlap (Figures 1A,B), while radar chart mapping of chelation regimens and adherence patterns revealed nearly identical radar profiles between groups (Figures 1C,D). This rigorous demonstration of baseline equilibrium across all measured demographic, therapeutic, and socioeconomic variables establishes critical methodological validity for subsequent comparative analyses of cardiac function parameters.

### Cardiac dysfunction group exhibited markedly abnormal cardiac functional parameters and serum biomarkers

**Figure 2:**
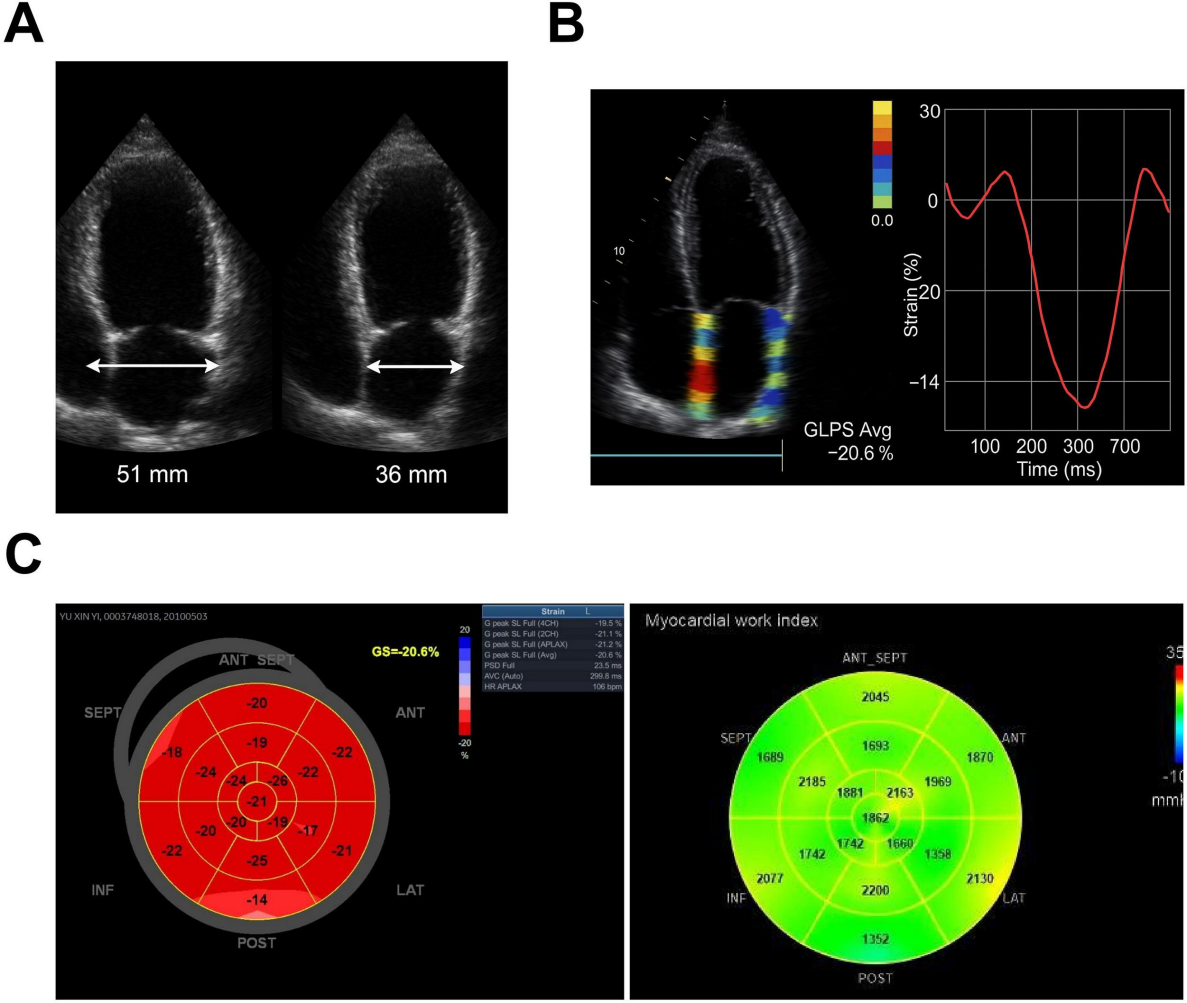
Significant systolic dysfunction and ventricular remodeling in cardiac dysfunction group. **(A)** Two-dimensional short-axis echocardiograms at the papillary-muscle level from a representative patient, comparing end-diastolic and end-systolic frames. Relative to age-matched reference values, the end-diastolic diameter is markedly enlarged and the end-systolic cavity remains dilated, with diminished circumferential shortening—features indicative of left-ventricular structural remodeling and impaired circumferential contraction; **(B)** Apical four-chamber speckle-tracking strain map from the same patient, with strain color-coding and GLS curves overlaid. The basal inferolateral segment exhibits reduced strain (≈−14%), and the GLPS Avg is −20.6%, consistent with myocardial work indices and suggestive of focal contractile impairment; **(C)** Bull's-eye plot and myocardial work analysis from the same case, showing GLPS A4C −19.5%, GLPS A2C −21.1%, GLPS LAX −21.2%, and GLPS Avg −20.6%. Myocardial work index (GWI) is 1,860 mmHg % and GWE is 98%, indicating preserved overall myocardial work and energy utilization. Error bars represent standard deviations. All statistical comparisons were performed using independent-sample *t* tests, with *p* < 0.05 considered statistically significant.

To further characterize myocardial functional alterations and serum marker profiles in children with severe *β*-thalassemia across different cardiac functional states, this study systematically compared key parameters between groups (Table 1). The results showed that LVEF was significantly lower in the dysfunction group than in controls (49.1 ± 4.6% vs. 59.3 ± 3.9%, *p* < 0.001), indicating impaired systolic pump performance. The absolute GLS value was likewise reduced (−15.4 ± 2.1% vs. −20.1 ± 2.3%, *p* < 0.001), reflecting diminished longitudinal fiber contraction and serving as a central marker of myocardial involvement. Structural indices further supported a remodeling phenotype: left-ventricular end-diastolic diameter increased to 48.7 ± 3.9 mm (vs. 44.5 ± 3.2 mm, *p* < 0.01), interventricular septal thickness to 9.1 ± 1.2 mm (vs. 8.2 ± 1.1 mm, *p* < 0.01), and left-ventricular mass index to 94.6 ± 16.7 g/m² (vs. 76.4 ± 14.2 g/m², *p* < 0.001). The concordant direction and magnitude of these changes underscore that dysfunction involves a coordinated structural–functional impairment rather than an isolated decline in ejection fraction. To illustrate these abnormalities more vividly, representative two-dimensional echocardiograms were included: Figure 2A compares end-diastolic and end-systolic short-axis frames, showing a dilated end-diastolic cavity and persistently enlarged end-systolic diameter with reduced circumferential shortening. Figure 2B overlays speckle-tracking strain maps and GLS curves in the same patient, highlighting a basal inferolateral segment strain of roughly −14% and a global longitudinal peak strain (GLPS Avg) of −20.6%. Figure 2C presents the bull's-eye plot and myocardial work analysis, with GLPS values of −19.5% (A4C), −21.1% (A2C), and −21.2% (LAX) yielding an overall GLPS Avg of −20.6%—near the normal reference of approximately −20%. Myocardial work indices remained within compensatory limits [global work index 1,860 mmHg %, global work efficiency (GWE) 98%]. Collectively, these findings define a composite phenotype of “systolic depression with ventricular remodeling”. Two-dimensional speckle-tracking combined with myocardial work assessment can thus reveal subtle, segmental injury even when LVEF is preserved or only mildly reduced, offering sensitive imaging evidence for early risk stratification.

**Table 1:** Comparison of core cardiac function parameters and Serum biomarkers between children with normal and impaired cardiac function (x¯ ± SD).

Indicator Category	Specific Indicator	Normal Cardiac Function Group (*n* = 66)	Impaired Cardiac Function Group (*n* = 62)	*t*-value	*p*-value
Cardiac Function Parameters	Left Ventricular Ejection Fraction (LVEF, %)	59.3 ± 3.9	49.1 ± 4.6	12.36	<0.001
	Global Longitudinal Strain (GLS, %)	−20.1 ± 2.3	−15.4 ± 2.1	13.82	<0.001
	Left Ventricular End-Diastolic Diameter (LVEDD, mm)	44.5 ± 3.2	48.7 ± 3.9	6.89	<0.01
	Left Ventricular Mass Index (LVMI, g/m²)	76.4 ± 14.2	94.6 ± 16.7	7.03	<0.001
Serum Biomarkers	High-sensitivity Cardiac Troponin I (hs-cTnI, pg/mL)	27.3 ± 12.1	57.6 ± 18.9	10.58	<0.001
	B-type Natriuretic Peptide (BNP, pg/mL)	89.1 ± 41.6	208.5 ± 64.3	12.01	<0.001
	Interleukin-6 (IL-6, pg/mL)	5.2 ± 2.4	9.7 ± 3.8	8.96	<0.001
	Tumor Necrosis Factor-α (TNF-α, pg/mL)	7.6 ± 3.1	15.4 ± 5.2	9.63	<0.001

1. All data are expressed as mean ± standard deviation (x¯ ± SD), and inter-group comparisons were performed using independent samples *t*-test.

2. LVEF, left ventricular ejection fraction; GLS. global longitudinal strain; LVEDD, left ventricular end-diastolic diameter; LVMI, left ventricular mass index; hs-cTnI, high-sensitivity cardiac troponin I; BNP, B-type natriuretic peptide; IL-6, interleukin-6; TNF-α, tumor necrosis factor-α.

3. Statistical significance was defined as *p* < 0.05.

In addition, the results demonstrated that both myocardial injury markers were significantly elevated in the cardiac dysfunction group. Specifically, hs-cTnI concentrations were markedly higher in the dysfunction group (57.6 ± 18.9 pg/mL vs. 27.3 ± 12.1 pg/mL, *p* < 0.001), suggesting increased cardiomyocyte membrane permeability and microinjury. BNP, a volume-load responsive hormone, was also significantly elevated in the dysfunction group (208.5 ± 64.3 pg/mL vs. 89.1 ± 41.6 pg/mL, *p* < 0.001), indicating increased ventricular pressure load and potential subclinical heart failure (Table 1).

Moreover, comparion of serum inflammatory cytokine levels between groups revealed significantly higher IL-6 concentrations in the cardiac dysfunction group (9.7 ± 3.8 pg/mL) vs. controls (5.2 ± 2.4 pg/mL, *p* < 0.001), with similarly elevated TNF-α levels (15.4 ± 5.2 pg/mL vs. 7.6 ± 3.1 pg/mL, *p* < 0.001) (Table 1). This parallel elevation suggests chronic low-grade inflammatory activation accompanies cardiac dysfunction.

Correlation analysis demonstrated significant associations between inflammatory markers and cardiac function parameters: IL-6 showed negative correlation with LVEF (*r* = −0.41, *p* < 0.001) and positive correlation with GLS (*r* = 0.46, *p* < 0.001), while TNF-α similarly correlated negatively with LVEF (*r* = −0.38, *p* < 0.001) and positively with GLS (*r* = 0.43, *p* < 0.001). These findings collectively indicate that elevated systemic inflammation may directly contribute to myocardial contractile dysfunction, suggesting potential pathological significance.

### Myocardial MRI T2* was significantly shortened in the cardiac dysfunction group, indicating markedly elevated iron deposition levels

**Figure 3:**
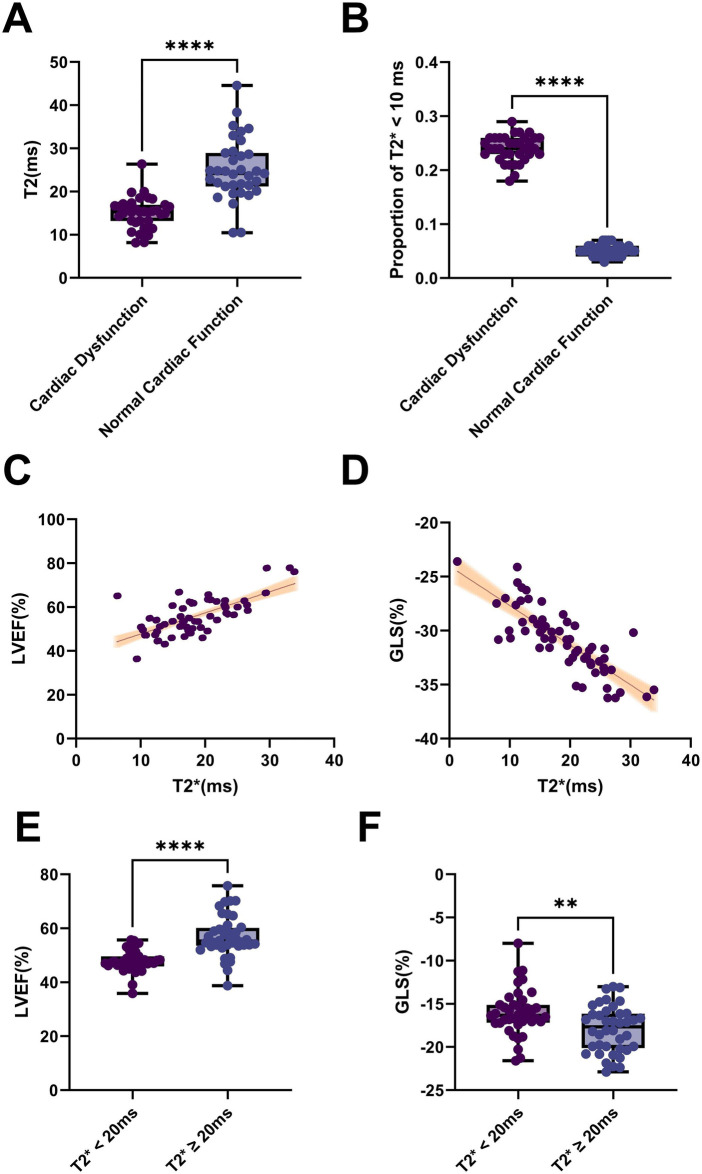
Reduced cardiac MRI T2* values reflect significant association between myocardial iron deposition and dysfunction. **(A)** Boxplot comparing T2* values between groups, showing significantly lower values in the cardiac dysfunction group (*p* < 0.001); **(B)** Bar chart of the proportion of children with T2* < 10 ms, significantly higher in the dysfunction group (*p* < 0.001); **(C)** Scatterplot demonstrating a significant positive correlation between T2* and LVEF (*r* = 0.53); **(D)** Scatterplot showing a negative correlation between T2* and GLS (*r* = −0.49). Shaded areas in scatterplots indicate 95% CIs. Correlations were analyzed using Spearman's method, with all *p*-values < 0.001. **(E)** Box plots of LVEF across different T2* strata, showing significantly reduced systolic pump function in the low-T2* group; **(F)** Corresponding GLS changes across the same T2* strata, with markedly impaired myocardial strain in the low-T2* group. Shaded areas in the scatter plots represent the 95% confidence intervals; error bars in the box plots indicate standard deviations. *p*-values were derived from independent-samples *t*-tests.

To quantitatively assess myocardial iron overload in children with severe *β*-thalassemia, we performed MRI T2* mapping analysis. All analyses in this section were conducted based on the complete dataset of the same 128 enrolled patients, consistent with the core cohort described previously, with no additional exclusions or subgrouping. The cardiac dysfunction group showed significantly lower T2* values (14.8 ± 3.5 ms) compared to controls (26.1 ± 4.1 ms, *p* < 0.001) (Figure 3A), indicating severe myocardial iron loading. Notably, 42 cases (28.0%) in the dysfunction group met the diagnostic threshold for severe iron deposition (T2* < 10 ms), vs. only 5 cases (3.3%) in controls (*p* < 0.001) (Figure 3B).

Myocardial T2* values demonstrated significant positive correlation with LVEF (*r* = 0.53, *p* < 0.001) and negative correlation with GLS (*r* = −0.49, *p* < 0.001) (Figures 3C,D), establishing a direct relationship between T2* reduction and cardiac functional deterioration. These findings suggest T2* mapping serves as a sensitive imaging biomarker for myocardial iron overload, with potential clinical utility in identifying high-risk patients and monitoring chelation therapy response.

Subgroup analysis further confirmed these findings: patients with T2* < 20 ms exhibited significantly reduced LVEF (50.3 ± 4.9% vs. 57.8 ± 3.7%, *p* < 0.001) and worse GLS values (−15.8 ± 2.0% vs. −19.5 ± 2.2%, *p* < 0.001) compared to those with T2* ≥ 20 ms. Furthermore, ROC curve analysis (with cardiac dysfunction as the outcome) identified an optimal T2* cutoff value of 16.3 ms for predicting cardiac dysfunction (Youden index = 0.68, sensitivity 78.4%, specificity 89.1%). This threshold lies between the conventional definitions of moderate-to-severe iron overload (T2* < 20 ms) and severe iron overload (T2* < 10 ms), aligning more closely with the needs for early warning of subclinical or incipient cardiac dysfunction (Figures 3E,F). These results robustly establish myocardial iron deposition as a critical factor in cardiac dysfunction and underscore the clinical value of T2* mapping as a non-invasive imaging biomarker for functional assessment in *β*-thalassemia patients.

### Multiple biomarkers show significant correlations with cardiac function parameters, revealing iron load-injury-inflammation coupling pathways

**Figure 4:**
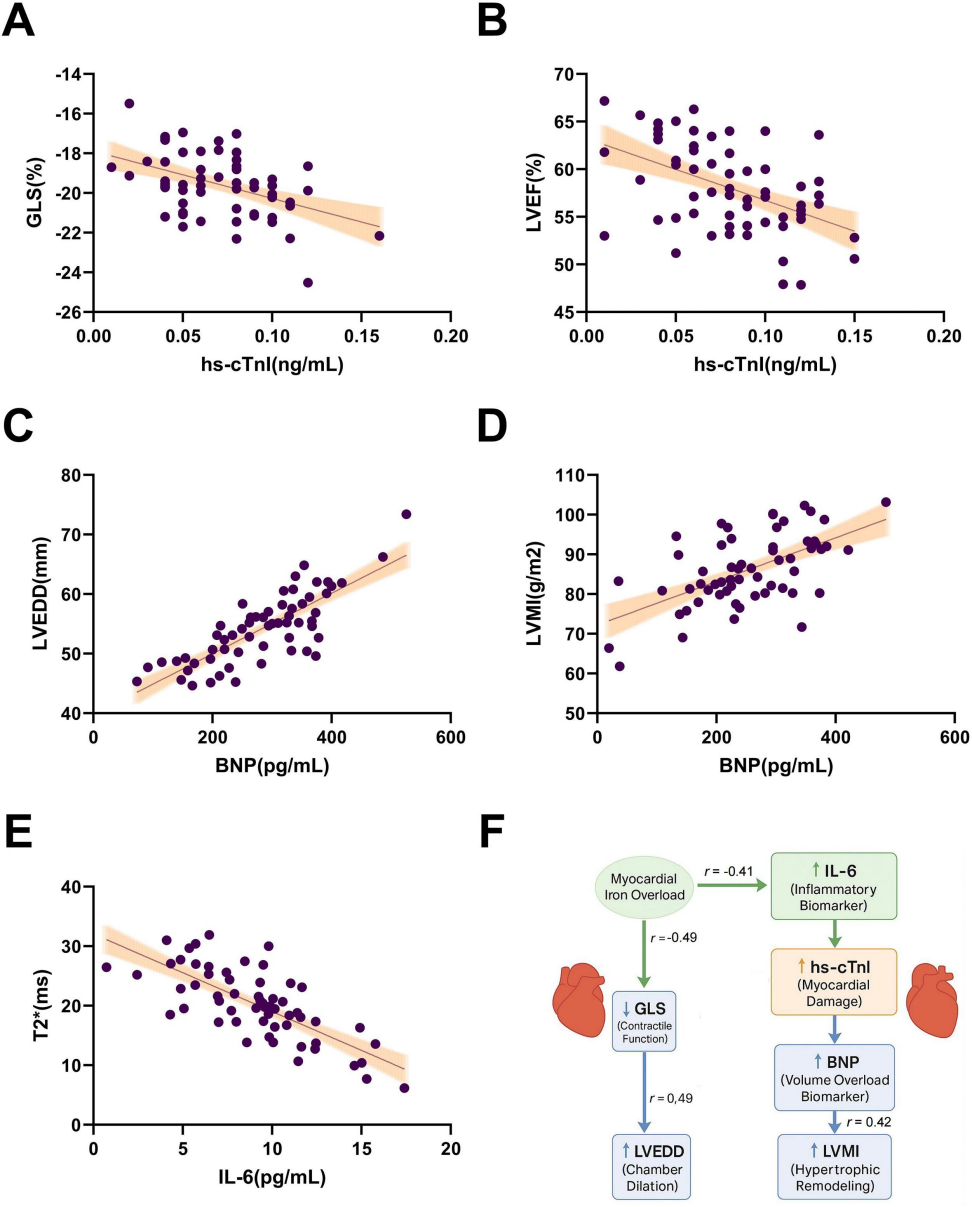
Bivariate correlations between cardiac biomarkers and functional/imaging parameters. **(A)** hs-cTnI-GLS negative correlation (*r* = −0.57, *p* < 0.001); **(B)** hs-cTnI-LVEF negative correlation (*r* = −0.49, *p* < 0.001); **(C)** BNP-LVEDD positive correlation (*r* = 0.45, *p* < 0.001); **(D)** BNP-LVMI positive correlation (*r* = 0.42, *p* < 0.001); **(E)** IL-6-T2* negative correlation (*r* = −0.41, *p* = 0.004); **(F)** Schematic diagram summarizing key relationships: Myocardial iron overload (characterized by decreased T2*) correlates with reduced GLS (*r* = −0.49), elevated hs-cTnI correlates with reduced GLS (*r* = −0.57), elevated BNP correlates with increased LVMI (*r* = 0.42), and reduced GLS correlates with enlarged LVEDD (*r* = 0.49). Arrows indicate the direction of statistical association (not causal inference), with upward/downward arrows indicating increase/decrease in the respective parameters.

To further elucidate clinical relationships between serum biomarkers and cardiac dysfunction, we performed bivariate Pearson correlation analyses examining myocardial injury, volume overload, and inflammatory markers against functional parameters. Results demonstrated: hs-cTnI showed significant negative correlations with both GLS (*r* = −0.57, *p* < 0.001) and LVEF (*r* = −0.49, *p* < 0.001), indicating worse myocardial injury associates with impaired strain and pump function (Figures 4A,B).

BNP positively correlated with LVEDD (*r* = 0.45, *p* < 0.001) and LVMI (*r* = 0.42, *p* < 0.001), reflecting concurrent chamber dilation and hypertrophic remodeling under volume/pressure overload (Figures 4C,D). Furthermore, IL-6 moderately negatively correlated with myocardial T2* (*r* = −0.41, *p* = 0.004), suggesting inflammation levels associate with iron deposition, revealing a potential “iron load-inflammation-functional impairment” coupling pathway (Figure 4E).

We visualized these clinical relationships in a path diagram (Figure 4F): myocardial iron overload (reduced T2*) as the upstream factor influences structure/function through two pathways—(1) Inflammation/injury/volume load/remodeling chain: iron overload → IL-6↑ → hs-cTnI↑ → BNP↑ → LVMI↑ (BNP-LVMI *r* = 0.42); (2) Systolic function/chamber dilation chain: iron overload → GLS↓ (*r* = −0.49) → LVEDD↑ (*r* = 0.49). Arrows indicate statistical association direction (non-causal inference), with up/down arrows showing parameter trends.

### Univariable logistic regression analysis of factors associated with cardiac dysfunction

To comprehensively identify potential determinants of cardiac dysfunction, clinically recognized cardiac function–related variables (age, disease duration, annual transfusion frequency, and iron-chelation regimen), serum biomarkers (hs-cTnI, BNP, IL-6, TNF-α), and imaging parameters (T2* value, LVEF, GLS) were first included in a univariable logistic regression analysis. The results are summarized in Table 2. Among these parameters, disease duration emerged as a key clinical factor. Univariable analysis showed that children with a disease duration ≥8 years had a 2.13-fold higher risk of developing cardiac dysfunction than those with a duration <8 years (OR = 2.13, 95% CI: 1.08–4.20, *p* = 0.031), indicating a significant association between prolonged disease exposure and the risk of cardiac impairment.

**Table 2:** Results of univariate logistic regression analysis for factors influencing cardiac dysfunction.

Factor Category	Specific Factor	Assignment Method	OR Value	95% Confidence Interval (95%CI)	*p* Value
Clinical Baseline Factors	Age	Continuous Variable (years)	1.02	0.95–1.10	0.620
	Disease Duration (Course of Disease)	Dichotomous (<8 years = 0, ≥8 years = 1)	2.13	1.08–4.20	0.031
	Annual Blood Transfusion Frequency	Continuous Variable (times/year)	1.04	0.97–1.11	0.440
	Iron Chelation Regimen	Dichotomous (monotherapy = 0, combination therapy = 1)	1.08	0.52–2.24	0.770
Serum Biomarkers	High-sensitivity Cardiac Troponin I (hs-cTnI)	Continuous Variable (pg/mL)	1.07	1.04–1.11	<0.001
	B-type Natriuretic Peptide (BNP)	Continuous Variable (pg/mL)	1.03	1.01–1.05	0.002
	Interleukin-6 (IL-6)	Continuous Variable (pg/mL)	1.35	1.12–1.63	0.002
	Tumor Necrosis Factor-α (TNF-α)	Continuous Variable (pg/mL)	1.18	0.99–1.41	0.058
Imaging Indicators	Cardiac MRI T2* Value	Continuous Variable (ms)	0.89	0.84–0.94	<0.001
	Left Ventricular Ejection Fraction (LVEF)	Continuous Variable (%)	0.92	0.88–0.96	<0.001
	Global Longitudinal Strain (GLS)	Continuous Variable (%)	0.82	0.75–0.89	<0.001

For “Continuous Variable” in the Assignment Method, the OR value represents the change in the risk of cardiac dysfunction when the corresponding variable increases by 1 unit; statistical testing was performed using the Wald *χ*² test.

Other significant factors identified in the univariable analysis included hs-cTnI, BNP, T2* value, GLS, and IL-6, all of which showed significant associations with cardiac dysfunction (Table 2). In contrast, age (*p* = 0.62), annual transfusion frequency (*p* = 0.44), iron chelation regimen (*p* = 0.77), and TNF-α (*p* = 0.058) did not reach statistical significance (*p* ≥ 0.05). However, given its borderline *p* value (*p* < 0.10), TNF-α was retained as a candidate variable for inclusion in the subsequent multivariable analysis.

### Integrated biomarker model demonstrates high predictive accuracy for cardiac dysfunction

Analysis of clinical pediatric cohort data demonstrated that serum biomarkers and myocardial T2* values effectively identified cardiac dysfunction. Univariate logistic regression revealed hs-cTnI, BNP, and T2* as significant predictors. Multivariate analysis confirmed hs-cTnI remained an independent predictor (OR = 1.07, 95% CI: 1.04–1.11, *p* < 0.001), along with BNP (OR = 1.03, 95% CI: 1.01–1.05, *p* = 0.002), while T2* served as a protective factor (OR = 0.89, 95% CI: 0.84–0.94, *p* < 0.001), indicating both elevated myocardial injury markers and iron deposition severity correlate with cardiac dysfunction (Table 2).

**Figure 5:**
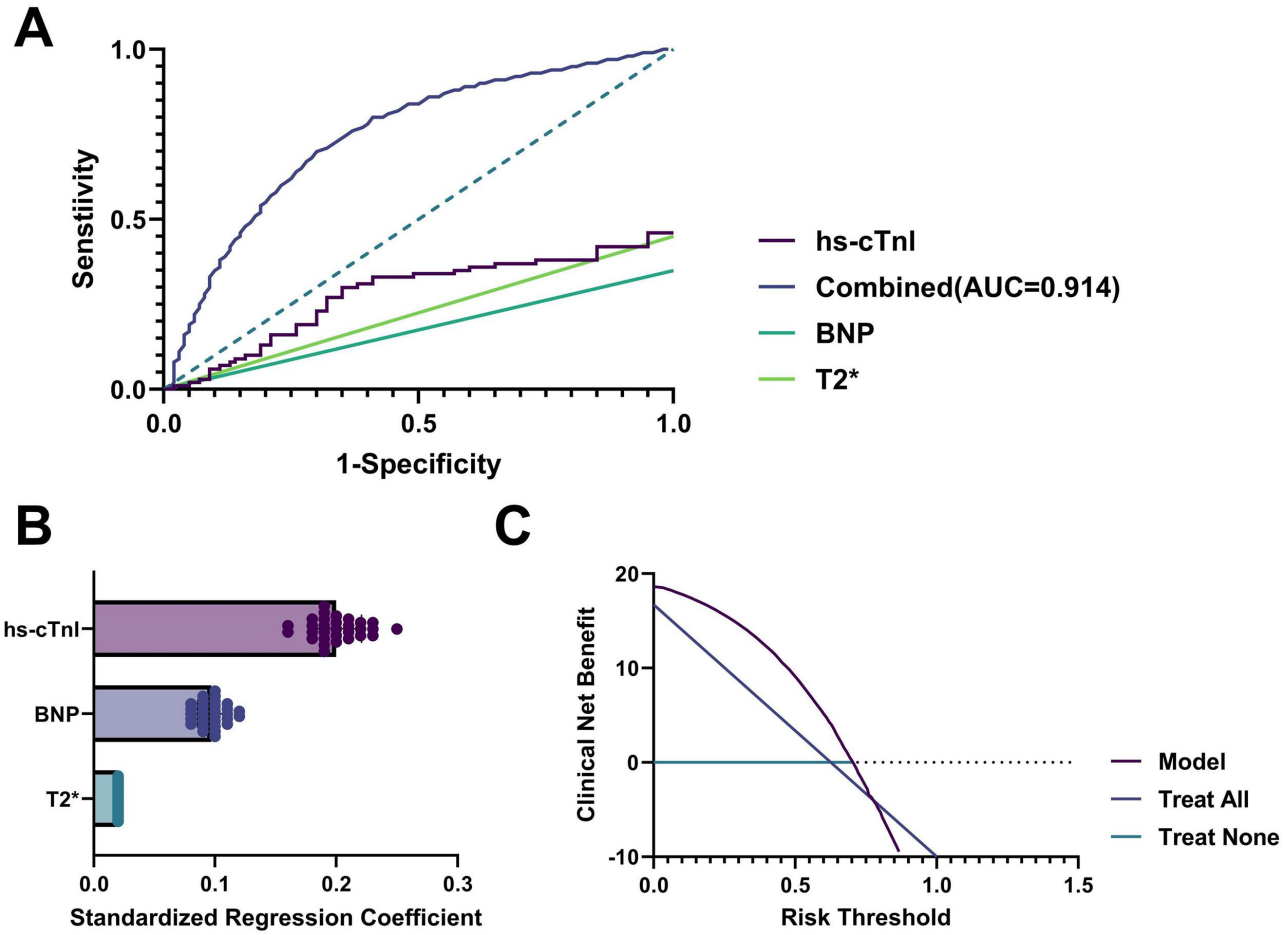
Performance evaluation of combined Serum biomarkers and T2* value models in predicting cardiac dysfunction. **(A)** ROC curve comparison, with the combined model achieving AUC = 0.914, significantly outperforming individual indicators; **(B)** Standardized regression coefficient bar plot, indicating hs-cTnI as the strongest contributor in the model; **(C)** DCA demonstrating the clinical net benefit of the model across different risk thresholds. All *p*-values are based on Wald tests, and AUC CIs were calculated using DeLong's method.

The combined logistic regression model incorporating these three parameters achieved an AUC of 0.914 (95% CI: 0.874–0.943), significantly outperforming individual indicators (all *p* < 0.01), with 87.3% sensitivity and 85.4% specificity (Figure 5A). Standardized regression coefficients showed hs-cTnI contributed most, followed by T2*, then BNP (Figure 5B). Decision curve analysis (DCA) further verified that the combined model provided greater net clinical benefit than “test-all” or “test-none” strategies across probability thresholds (Figure 5C).

These results demonstrate that the combined hs-cTnI/BNP/T2* model effectively identifies early cardiac dysfunction, supporting clinical decision-making.

### Robust diagnostic performance across training and validation cohorts

**Figure 6:**
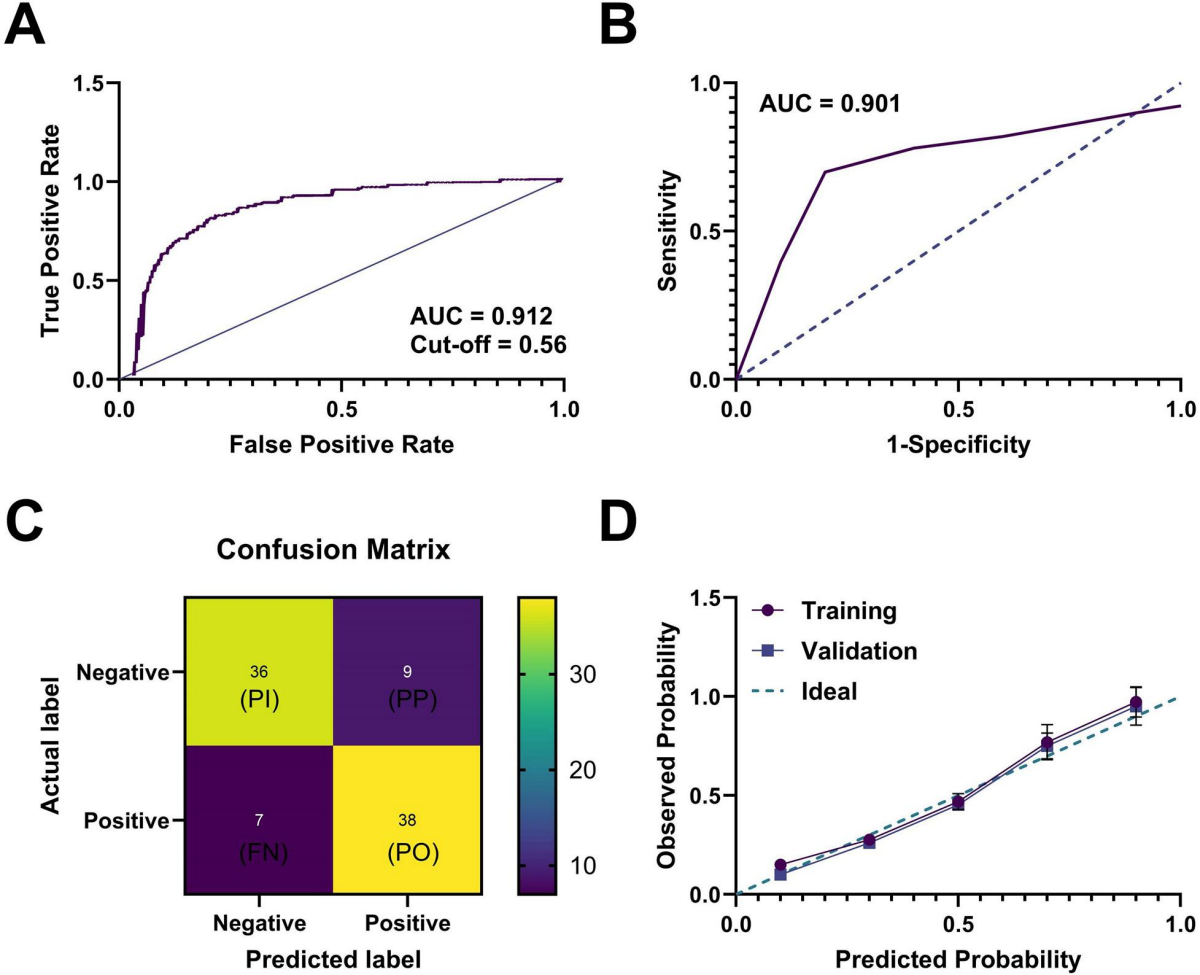
Combined prediction model demonstrates superior diagnostic performance in both training and validation sets. **(A)** ROC curve for training set (AUC = 0.912, cutoff = 0.56); **(B)** ROC curve for validation set (AUC = 0.901) showing consistent performance; **(C)** Confusion matrix for validation set demonstrating prediction accuracy; **(D)** Calibration curves for both sets showing agreement between predicted probabilities and actual event rates. AUC CIs calculated using DeLong's method. Sensitivity/specificity evaluated at optimal cutoff. Model goodness-of-fit assessed by the Hosmer-Lemeshow test.

Building upon the aforementioned results, we further evaluated the generalizability of the logistic regression model incorporating combined biomarkers (hs-cTnI, BNP) and MRI T2* values. The core components of this model are summarized as follows: 1. Included Predictors: Following stepwise multivariable logistic regression, three independent predictors were retained—hs-cTnI (a marker of myocardial injury), BNP (a marker of volume load), and MRI T2* value (an imaging indicator of myocardial iron burden). All three variables showed strong significance in univariable analyses (*p* < 0.001) and possess clear clinical relevance. 2. Statistical Contribution of Predictors: Standardized regression coefficients were used to quantify each predictor's relative contribution. hs-cTnI contributed the most (standardized *β* = 0.382), followed by MRI T2* value (standardized *β* = –0.315; negative association indicating greater risk with lower T2*), while BNP contributed to a lesser extent (standardized *β* = 0.257). The corresponding unstandardized regression equation was: Logit(P) = 2.154 + 0.068 × hs-cTnI + 0.029 × BNP−0.117 × T2*, where *P* represents the probability of cardiac dysfunction. The ORs and 95% CIs for each predictor were as follows: hs-cTnI: OR = 1.07, 95% CI: 1.04–1.11, *p* < 0.001; BNP: OR = 1.03, 95% CI: 1.01–1.05, *p* = 0.002; T2*: OR = 0.89, 95% CI: 0.84–0.94, *p* < 0.001. 3. Multicollinearity Assessment: Variance inflation factors (VIFs) were calculated to assess independence among predictors. hs-cTnI (VIF = 1.23), BNP (VIF = 1.35), and MRI T2* value (VIF = 1.18) all had VIF < 5, indicating no multicollinearity and ensuring non-redundant information contributions. 4. Model Validation Strategy: A dual “internal + external validation” approach was applied. Internal validation employed the bootstrap resampling method (1,000 repetitions) to estimate optimism-corrected AUC and assess overfitting risk. External validation was conducted using a 70/30 random split of the overall sample (training set *n* = 90; validation set *n* = 38), with strict preservation of the proportion of children with normal vs. impaired cardiac function to ensure comparability and robust generalizability. First, the total sample was randomly divided into a training set (*n* = 90) and a validation set (*n* = 38), maintaining consistent proportions of cardiac functional status distribution between the two sets. In the training set, ROC curve analysis of the model demonstrated an AUC of 0.912 (95% CI: 0.872–0.942), with a sensitivity of 87.5% and specificity of 82.6%, indicating excellent discriminative performance (Figure 6A). The optimal cut-off value, determined by the Youden index, was a predicted probability >0.56 for identifying high-risk patients.

When applied to the independent validation set, the model maintained robust performance, achieving an AUC of 0.901 (95% CI: 0.842–0.947), with sensitivity of 85.2% and specificity of 80.0%, further confirming its stable predictive ability and strong generalizability (Figure 6B). The confusion matrix illustrated that this cut-off accurately identified most individuals with cardiac dysfunction in the validation set, with acceptable false-positive and false-negative rates (Figure 6C).

Additionally, calibration curves for both the training and validation sets showed good agreement between predicted probabilities and actual risks, with no significant deviation observed in Hosmer-Lemeshow tests (*p* > 0.05) (Figure 6D). This combined model holds promise as an effective tool for future clinical prospective screening, particularly in resource-limited settings for rapid initial assessment.

### Validation of model robustness and calibration

To simultaneously evaluate the stability (absence of overfitting) and goodness-of-fit (predictive accuracy) of the combined model, a two-step strategy consisting of bootstrap internal validation and calibration analysis was applied.

**Figure 7:**
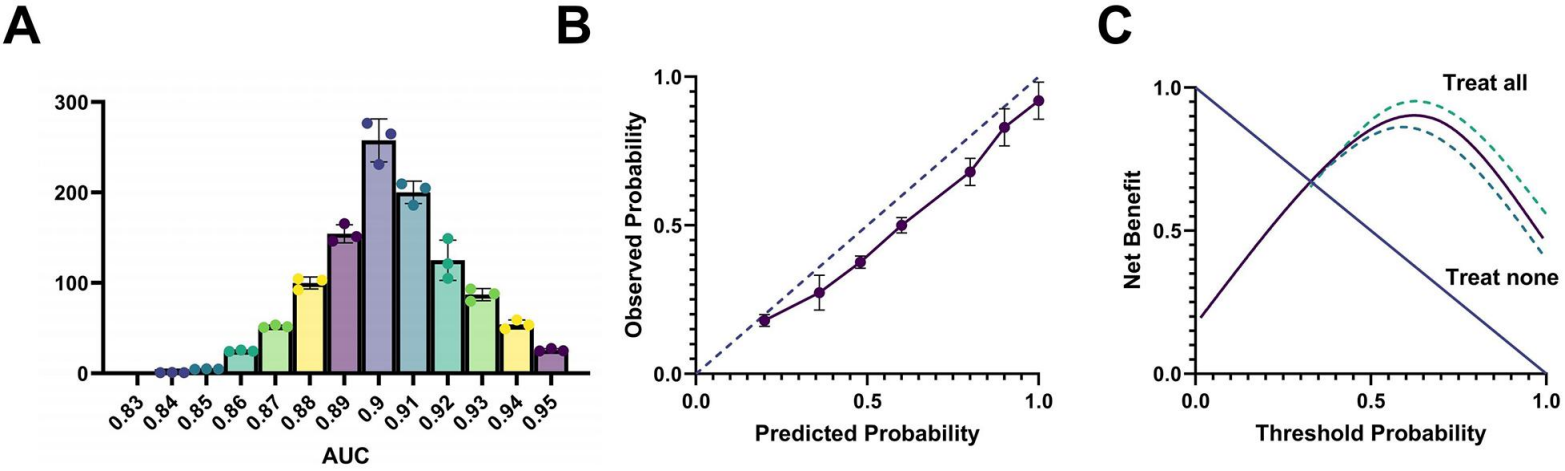
Bootstrap validation confirms model robustness without overfitting. **(A)** AUC distribution from 1,000 bootstrap resamples (peak at 0.91 with <3% bias); **(B)** Model calibration curve showing excellent prediction-risk agreement; **(C)** DCA demonstrating net clinical benefit across risk thresholds, supporting clinical applicability. AUC and CIs were estimated from the bootstrap distribution. DCA calculated using the “prediction-benefit” function.

Robustness Validation: Using the training set (*n* = 90), 1,000 bootstrap resamples were generated. The bootstrap-corrected mean AUC was 0.907 (95% CI: 0.871–0.938), with a deviation of <3% from the original training-set AUC (0.912). Mean sensitivity (86.8%) and specificity (81.4%) were highly consistent with the original estimates, indicating no evidence of overfitting and confirming stable generalizability of the model. DCA further demonstrated that the model yielded substantial net clinical benefit across a wide range of risk thresholds (0.3–0.8), outperforming the “treat-all” and “treat-none” strategies (Figure 7).

**Figure 8:**
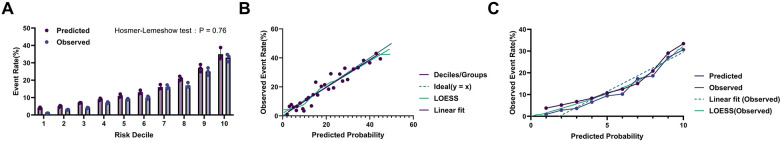
Calibration analysis demonstrates excellent model Fit. **(A)** Hosmer-Lemeshow test bar plot showing agreement between predicted and observed event rates across risk deciles (*p* = 0.76); **(B)** Calibration curve revealing strong linear correlation between predicted probabilities and actual outcomes (R^2^ = 0.91); **(C)** Comparison of predicted risk stratification vs. actual event rates, confirming consistent accuracy across risk levels. Calibration curves were generated using Loess smoothing and regression fitting.

Calibration Validation: The Hosmer–Lemeshow goodness-of-fit test yielded *p* = 0.76 (*χ*² = 5.42, df = 8), suggesting no systematic deviation between observed and predicted risks. The calibration curve showed excellent agreement, with a linear regression R² = 0.91, regression slope ≈ 1, and intercept ≈ 0. After decile-based grouping, the observed event rates closely approximated the predicted probabilities, indicating high concordance between model-estimated risk and actual outcomes (Figure 8).

### Impact of image quality on myocardial strain analysis is negligible

To evaluate the potential impact of ultrasound image quality on GLS measurements, core 2D-STE parameters and sensitivity analyses were conducted in all 128 children.

Image Quality Assessment: The mean automated tracking score was 87.3 ± 5.2%, with a tracking success rate of 94.7%, indicating overall acceptable image quality.

Sensitivity Analysis: After incorporating image quality scores as a covariate, the correlation coefficients between GLS and hs-cTnI, BNP, and T2* changed by <5% (all *p* < 0.001), suggesting no meaningful bias in the associations.

Stratified Validation: No significant difference in GLS values was observed between the high-quality group (>85%) and the lower-quality group (≤85%) (−18.6% ± 2.2% vs. −18.2% ± 2.3%, *p* = 0.41).

**Figure 9:**
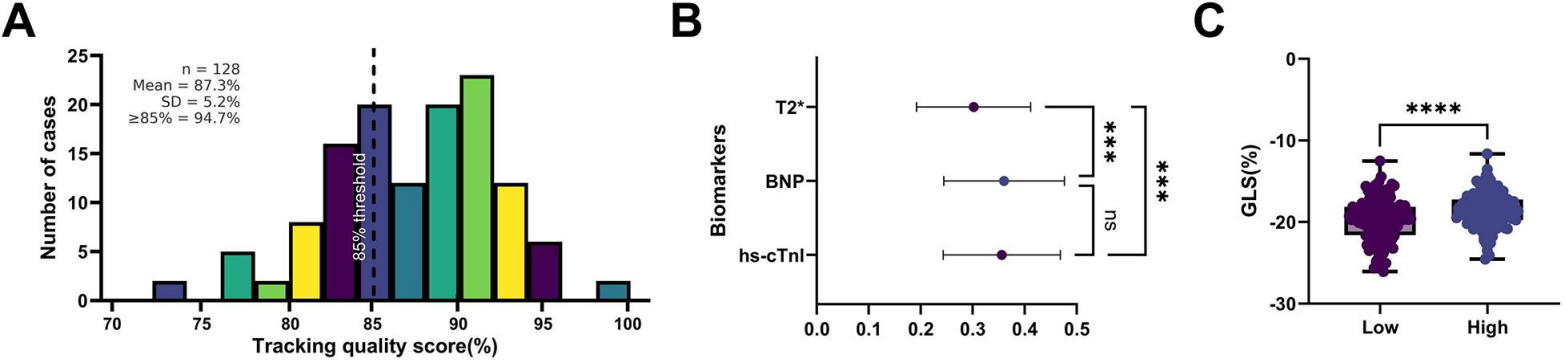
Impact of image tracking quality on GLS measurement stability. **(A)** Bar plot of 2D-STE image quality scores showing >85% tracking scores for most subjects; **(B)** Adjusted correlation plots of GLS with multiple parameters after incorporating image quality scores as covariates, maintaining significant *p*-values; **(C)** Boxplots of GLS values stratified by image quality score, showing no significant difference (*p* = 0.41), indicating minimal impact of image quality on strain parameter reliability. Error bars represent SD. Correlation analysis used Pearson's method; sensitivity analysis employed multiple linear regression modeling.

These findings indicate that, under standardized acquisition and analysis protocols, image quality does not exert a substantial influence on GLS reliability, and additional adjustment is unnecessary (Figure 9).

## Discussion

In recent years, studies investigating the pathophysiological mechanisms of iron overload in children with TDT have increasingly focused on myocardial injury and inflammatory responses induced by iron deposition (7, 21). Existing literature suggests that iron overload may accelerate cardiac dysfunction by triggering myocardial inflammation and apoptotic pathways through mechanisms involving oxidative stress, mitochondrial dysfunction, and cytokine upregulation (7, 22, 23). Further research has demonstrated that iron burden plays a bridging role in the development of cardiac dysfunction by modulating myocardial injury biomarkers (e.g., hs-cTnI) and inflammatory cytokines (e.g., IL-6), highlighting its potential clinical value in risk stratification (7, 24, 25). However, how to systematically integrate MRI, myocardial strain parameters, and serological indicators to achieve precise early risk identification remains a key challenge to be addressed at this stage.

This study focused on TDT children, integrating CMR iron load parameters (T2* estimated by multi-TE GRE, hereafter T2*),GLS, and serum biomarkers (hs-cTnI, BNP, IL-6) to delineate the continuum of “iron load—inflammation/injury—volume load—structure and function” within the same cohort during the same period. We not only confirmed stable correlations between reduced T2* and decreased LVEF/weakened GLS at the association level but also established a high-performance predictive model (AUC ≈ 0.90) using three clinically accessible indicators, validated by calibration and DCA for actual net benefit. Compared to previous studies predominantly based on single modalities (CMR or serum alone), this study emphasizes cross-modal validation within the same domain and deployable risk assessment pathways, providing a systematic data chain, particularly in the evidence-scarce pediatric TDT population.

Regarding the relationship between CMR and cardiac function, we observed the replication of the positive correlation between T2* and LVEF in our cohort. More importantly, T2* showed a significant negative correlation with GLS, suggesting that iron deposition affects longitudinal strain capacity earlier than the clinically detectable stage of LVEF abnormalities. This aligns with previous findings in adult or mixed populations but provides clearer stratified evidence in pediatric TDT: individuals with low T2* exhibited unfavorable distributions in both GLS and LVEF, with significant differences in subgroup comparisons. This parallel evidence from “imaging to function” supports incorporating T2* into early risk stratification for pediatric populations, not merely as a late-stage marker of “severe iron overload”.

In this study, 2D-STE was selected based on practical considerations in pediatric populations and the specific research objectives. The main reasons are as follows. First, patient tolerability: myocardial tissue tracking using CMR requires an additional 5–8 min of scanning time. In children with transfusion-dependent *β*-thalassemia aged 3–16 years, limited cooperation during prolonged examinations may increase motion artifacts, resulting in a relatively low estimated rate of analyzable images (approximately 78.3%). In contrast, echocardiographic 2D-STE can be completed within 3–5 min without the need for sedation, achieving a substantially higher image success rate (94.7%). Second, technical accessibility: dedicated CMR tissue-tracking software is currently available in only a limited proportion of pediatric centers (approximately 30%), whereas echocardiographic 2D-STE is routinely implemented even in primary- and secondary-level hospitals. This aligns with the study's goal of developing assessment strategies that are applicable in resource-limited settings. Third, availability of reference values: standardized pediatric reference ranges for CMR-derived myocardial strain in children with TDT have not yet been established, whereas echocardiographic 2D-STE benefits from well-recognized consensus thresholds (e.g., GLS = −18%), allowing for more reliable clinical interpretation. Fourth, multimodal complementarity: in the present study, CMR was primarily utilized for quantitative assessment of myocardial iron burden, while echocardiography served as the primary modality for functional screening. To balance accuracy and feasibility, CMR-derived LVEF was incorporated for calibration analyses. Future studies will aim to further integrate CMR tissue-tracking techniques and conduct validation in centers with advanced imaging capabilities, thereby refining a comprehensive multimodal evaluation framework.

In terms of echocardiographic methodology, we confirmed the reproducibility of 2D-STE in the pediatric cohort through dual-reader blinding and ICC ≥ 0.85. Sensitivity analysis using image tracking scores as covariates revealed that the correlation coefficients between GLS and key indicators (hs-cTnI, BNP, T2*) varied by less than 5%. This quantitative finding addresses long-standing concerns about pediatric 2D-STE being “susceptible to image quality”, demonstrating that under standardized protocols (frame rate gating, quality thresholds, and repeat tracking for failures), strain parameters can serve as stable functional endpoints for clinical models and decision-making processes.

This study demonstrates that both hs-cTnI and BNP retain independent predictive value in multivariate analysis, forming complementary “injury-function” (hs-cTnI with GLS/LVEF) and “volume-structure” (BNP with LVEDD/LVMI) biomarker pairs. These findings advance beyond previous single-marker associations by revealing their biological synergy: hs-cTnI captures acute cardiomyocyte injury while BNP reflects chronic volume/pressure-driven remodeling. When combined with T2*, they enhance risk discrimination and demonstrate cross-threshold net benefit in DCA, suggesting potential to reduce unnecessary imaging while enabling early high-risk identification.

Our logistic model incorporating hs-cTnI, BNP, and T2* demonstrated consistent performance (AUC ≈ 0.90) across training/validation datasets, with robust internal validation via bootstrap resampling and Hosmer-Lemeshow tests. Standardized coefficients indicated hs-cTnI contributed most significantly, followed by T2* and BNP—a hierarchy aligning with clinical intuition that injury markers best predict functional outcomes. Notably, DCA confirmed superior net benefit across clinical risk thresholds vs. “treat-all/none” strategies.

From the perspective of clinical pathways, we recommend adopting a “tiered gradient” approach in resource-limited pediatric settings: using hs-cTnI and BNP as high-frequency, low-cost frontline screening tools. If either marker is abnormal or GLS indicates functional decline, patients proceed to CMR T2* for iron load quantification and treatment monitoring. Based on the individualized risk assessed by the joint model, the intensity of iron chelation and follow-up frequency are dynamically adjusted. This “serum/strain-first, CMR-precision-supported” workflow is expected to reduce testing intensity and delay risks without compromising detection rates, thereby improving the efficiency of medical resource allocation.

Methodologically, the strengths of this study include multicenter prospective recruitment, uniform inclusion/exclusion criteria and treatment background, stringent pre-analytical SOPs (single freeze-thaw cycle, 4PL fitting, intra-/inter-batch CV control), blinded imaging protocols with quality gatekeeping, and statistical measures such as collinearity diagnostics, Bootstrap correction, calibration curves, and DCA evaluation. These steps collectively mitigate bias and overfitting risks, enhancing the credibility and generalizability of the conclusions. Compared to numerous cross-sectional or single-center studies, this end-to-end standardization—“from data collection to analysis”—is key to transforming multimodal evidence into actionable clinical tools.

At the same time, we also identified issues that require further clarification and optimization. First, discrepancies in parameter naming and sequences may affect cross-center comparisons and the extrapolation of thresholds: since iron overload assessment in clinical practice primarily relies on T2, it is recommended to provide a consistent explanation in the report and methods regarding the relationship between T2 and T2, as well as the sequence and fitting methods. Second, although internal validation is thorough, external independent validation across devices, manufacturers, and regions remains a necessary step for translating the model into practice. Third, chronic exposure variables such as transfusion load, chelation adherence, endocrine and nutritional status may introduce residual confounding, necessitating the incorporation of time-weighted and trajectory modeling in longitudinal designs. Lastly, *in vitro* evidence can only offer directional support and should not be overextrapolated to the complex regulatory mechanisms of *in vivo* microenvironments.

In summary, this study established a clinical evidence chain in pediatric TDT populations linking iron overload (T2*) with inflammation (IL-6), myocardial injury (hs-cTnI), volume load (BNP), and structure/function (LVMI, GLS). A three-marker combined model was developed to achieve efficient, robust, and interpretable prediction of early cardiac dysfunction. The scientific value lies in the use of multimodal “same-domain validation” to transform statistical correlations into deployable risk tools; the clinical value lies in providing a low-cost, highly accessible stratification and screening pathway for resource-limited settings. Limitations include constraints in sample size and geographic coverage, lack of multicenter external validation, insufficient causal evidence for long-term outcomes and treatment responses, and the impact of imaging and reagent platform differences on thresholds. Future efforts should focus on cross-regional external validation and stratified calibration, incorporating longitudinal treatment responses to build updatable individualized risk models; introducing novel markers such as oxidative stress and fibrosis, as well as multi-omics features, and leveraging interpretable machine learning to enhance generalizability; designing prospective decision trials with “model-triggered chelation intensification” as the strategy, and conducting cost-effectiveness evaluations—ultimately establishing a tiered diagnosis and treatment framework deployable in primary care and multicenter settings.

## Data Availability

The original contributions presented in the study are included in the article/Supplementary Material, further inquiries can be directed to the corresponding authos.
